# Toward noninvasive precision: a meta-analysis of photoacoustic spectroscopy in breast cancer

**DOI:** 10.1007/s10103-025-04714-2

**Published:** 2025-10-27

**Authors:** Shashanka Puranika K, Manjunath B Malshetty, Manya Gireesh Mooliyil, Budheswar Dehury, Nirmal Mazumder

**Affiliations:** 1 Department of Bioinformatics, Manipal School of Life Sciences, Manipal Academy of Higher Education, Manipal, Karnataka 576104 India; 2 Department of Biophysics, Manipal School of Life Sciences, Manipal Academey of Higher Education, Manipal, Karnataka 576104 India

**Keywords:** Breast cancer diagnosis, Photoacoustic spectroscopy (PAS), Early breast cancer detection, Tumor heterogeneity

## Abstract

Breast cancer is one of the most common lethal cancers in the world. Standard diagnostic methods, particularly tissue biopsies, are invasive and often fail to detect tumor heterogeneity, leading to suboptimal treatment outcomes. This study aims to assess and evaluate the potential of photoacoustic imaging (PAI) and photoacoustic spectroscopy (PAS) as noninvasive technologies that replace conventional methods for diagnosing breast cancer with increased depth of tissue penetration, improved resolution, and molecular sensitivity. This meta-analysis introduces the principal advantages and limitations of the PAI and PAS technologies. It applies a statistical approach to assess diagnostic performance, sensitivity, and specificity in preclinical and experimental settings. Data from six relevant studies on PAS for breast cancer screening were summarized. The analysis revealed a pooled sensitivity of 84% and specificity of 96% to assess the diagnostic performance of the PAS/PAI technologies. The estimate of pooled logit-transformed diagnostic performance was 0.72 (95% CI: 0.71–0.72), equivalent to 84% pooled sensitivity and 96% specificity. The logit value reflects variance-stabilized analysis and cannot be interpreted as uncorrected diagnostic accuracy, and the average heterogeneity across studies was moderate (I^2^ = 51.0%). These results highlight the potential of the PAI and PAS as reliable and noninvasive diagnostic instruments with the ability for early detection as well as improved patient outcomes.

## Introduction

Breast cancer remains a major public health issue that continues to affect patients globally and is the most common form of cancer in women [1]. In 2022 alone, more than 2.3 million new breast cancer cases occurred among women, with almost 670,000 of these patients sadly perishing in the same year [2–4]. These statistics reflect the global public health and social burden of breast cancer. In addition to the individual losses of patients and their families, breast cancer also imposes a massive strain on health systems worldwide. The cost of the treatment, hospital stay, and long-term care necessary for patients has substantially increased over the years. With increased life expectancy and the value of early detection of the disease becoming a factor, it is estimated that these alarming numbers will continue to grow exponentially in the coming decades.

By 2050, experts predict that the number of new breast cancer diagnoses could increase to 3.2 million annually, with the number of deaths possibly reaching 1.1 million [5]. There is need to improve cancer research, particularly in diagnostic and treatment strategies. For a long time, managing cancer has relied on catching it early when treatments are more likely to work. A key method for this is tissue biopsy, which helps doctors determine if cancer is present. While these biopsies have been helpful, they also have a fair share of issues. They can be invasive, often causing patients discomfort, and the whole process can take longer than we would like. Additionally, because biopsies involve only tiny tissue samples, there is a good chance that important details about the tumor can be missed.

To this point, the most widespread and highly recommended test for diagnosing this disease is referred to as tissue biopsy, which is further investigated through careful examination. This process provides physicians with vital and crucial information on the grade, type, and molecular attributes of the tumor, all of which serve as imperative factors in determining the best and most effective type of treatment to be administered in the future [6]. Nevertheless, the process of conducting biopsies is invasive and expensive. Furthermore, there is also a risk of sampling errors, especially when tumors have heterogeneous attributes on their entire surface. Tumours are dynamic and may change over time, showing differences depending on the area from which they are sampled, thereby making it difficult to have a full understanding in the case where only a portion of the tissue is investigated. Biopsies are painful to patients and are not always well suited for repeated monitoring or when tumors are in hard-to-reach areas. After all, tumors are not uniform; they can vary widely in size and make up between different people and the same person over time. When we look at only small sections of a tumor, we might miss crucial bits of information, leading to incorrect diagnoses and less effective treatments [7] To address these issues, researchers have investigated noninvasive diagnostic devices that can provide real-time information about tumor biology without the use of conventional biopsies. One of the devices that has been proposed is liquid biopsy. This process enables physicians to examine blood samples for tumor markers such as circulating tumor DNA (ctDNA), circulating tumor cells (CTCs), and other material that can provide us with similar information about the tumor as a tissue biopsy [8–10]. The notable advantage of liquid biopsy is that it is able to monitor cancer progression, determine whether the tumor is becoming resistant to treatment, and test how effective the treatment is, all from a single blood draw [11, 12].

Notably, groundbreaking imaging technology advancements have revolutionized the art and science of noninvasive diagnosis [12, 13]. The advanced techniques used for imaging are now capable of delivering not only anatomical information but also critical functional and molecular information about body systems. Imaging technologies such as MRI, PET, and ultrasound have become unavoidable in cancer detection, monitoring, and tracking patients at various stages [14]. Nonetheless, it is important to recognize that these imaging technologies also possess frailties; they are too costly, subject patients to ionizing radiation, or, in certain cases, are not detailed enough to spot early-stage tumors with accuracy. Recent innovations have shown that photoacoustic imaging (PAI) has emerged as a promising new imaging technology that combines the high resolution of light-based imaging with the deep penetration ability of ultrasound technology. The new process works by shining a pulsed beam of laser light onto the tissue, which selectively absorbs specific materials such as hemoglobin, causing the tissue to swell and then produce sound waves that can be processed into high-resolution images.PAI does not just show what tissues look like; it helps measure vital functions such as blood flow and oxygen levels, which are key indicators of tumor growth. This technique is attractive because it uses natural markers in the body, such as hemoglobin, which means that patients can avoid invasive procedures [13, 14]. While PET has molecular-level imaging capability and MRI has superior soft tissue contrast, the two tests are costly and involve irradiating patients with ionizing radiation. While very ubiquitous and nonionizing, ultrasound is limited by inferior contrast resolution and operator dependency. To overcome these drawbacks, PAS and PAI offer high-resolution molecular contrast with cost-effectiveness and safety. However, their availability in low-resource environments may be limited by their requirements for laser-based systems and dedicated detection setups. Toward increased clinical application, future development should aim to make systems affordable, portable, and user friendly.

Another significant photoacoustic extension of photoacoustic imaging is photoacoustic spectroscopy (PAS), which is gaining popularity in the medical field [17]. This method merges optics with the basics of thermodynamics. When biological tissues are exposed to light, some molecules absorb that light energy. This tiny increase in temperature creates sound waves that can be picked up, providing valuable information about the state of the tissues. PAS demonstrates superior tissue penetration compared to traditional optical methods such as fluorescence and absorption imaging. However, it does not necessarily outperform other optical modalities in detecting subtle biochemical changes. It can even reveal the difference between tumor tissues and healthy tissues by detecting variations in blood oxygen levels, which results in different sound signatures. Because PAS can detect this phenomenon, it shows real promise for early cancer detection and tracking how effective treatments are [15–18] There are issues with PAS signals being affected by surrounding tissues and concerns about how well the devices can work with various types of tissuesFortunately, scientists are working on improving device designs, tweaking frequency settings, and even using artificial intelligence to enhance signal quality and lower noise. One of the major advantages of PAS is its potential to reach people in underserved areas, as well as the widespread use of smartphones, as well as the decreasing technological costs, which could help more patients obtain an early diagnosis, which is crucial for better outcomes. It is essential to monitor advancements in noninvasive diagnostic tools such as PAS in breast cancer patients continuously. After looking closely at multiple studies, we can obtain a clearer idea of the effectiveness of PAS and how it can be best used in clinical settings.

This analysis aims to fill a gap in research by closely examining how well PAS works in the diagnosis of breast cancer. Unlike other types of reviews that tell you what studies found, this analysis combines the findings from different studies to provide a clearer picture of how PAS performs. We want to help doctors, researchers, and those making healthcare decisions better understand how PAS fits into cancer diagnosis. With technology improving all the time, PAS is moving beyond being just a new idea and is starting to show real potential to help catch breast cancer early and tailor treatments to individual patients. This is important because early detection can help to improve breast cancer acre for everyone. However, we believe that understanding PAS better will help improve breast cancer care for everyone.

## Meta-Analysis

The meta-analysis presented here was performed following a standardized methodology to ensure a transparent, reproducible, and scientifically rigorous synthesis of the literature. This approach is in accordancewith internationally recognized standards, including the PRISMA (Preferred Reporting Items for Systematic Reviews and Meta-Analyses) 2020 guidelines [19, 20]. The entire process encompasses several critical stages: defining inclusion and exclusion criteria, conducting a comprehensive literature search, screening and selecting relevant studies, performing quantitative synthesis via appropriate statistical models, and evaluating the results via detailed visualization and heterogeneity analysis.

Any meta-analysis begins with the careful resolution of eligibility criteria to select the required papers. These criteria ensure that only methodologically correct and clinically relevant studies are included in the synthesis. The inclusion criteria are typically based on predefined characteristics such as the type of study design (e.g., randomized controlled trials, cohort studies), target population (e.g., age, sex, or specific conditions such as pregnancy-associated breast cancer), type of diagnostic modality (in this case, PAS or PAI), and clearly defined diagnostic performance outcomes (e.g., sensitivity, specificity, diagnostic odds ratio) [21]. Exclusion criteria eliminate studies that may introduce bias or inconsistencies, including duplicate publications, review articles, editorials, studies lacking quantitative data, and those with methodological flaws.

### Search strategy and study selection

A systematic and exhaustive literature search was performed via major biomedical databases such as PubMed, EMBASE, and Scopus [22]. The search strategy included a combination of Medical Subject Headings (MeSH) and free-text terms related to photoacoustic imaging and breast cancer diagnostics. The search terms were applied across all fields and filtered for English-language, peer-reviewed articles published in full text. In addition to database searches, reference lists of identified studies and relevant reviews were manually examined to identify additional eligible studies.

**Fig. 1:**
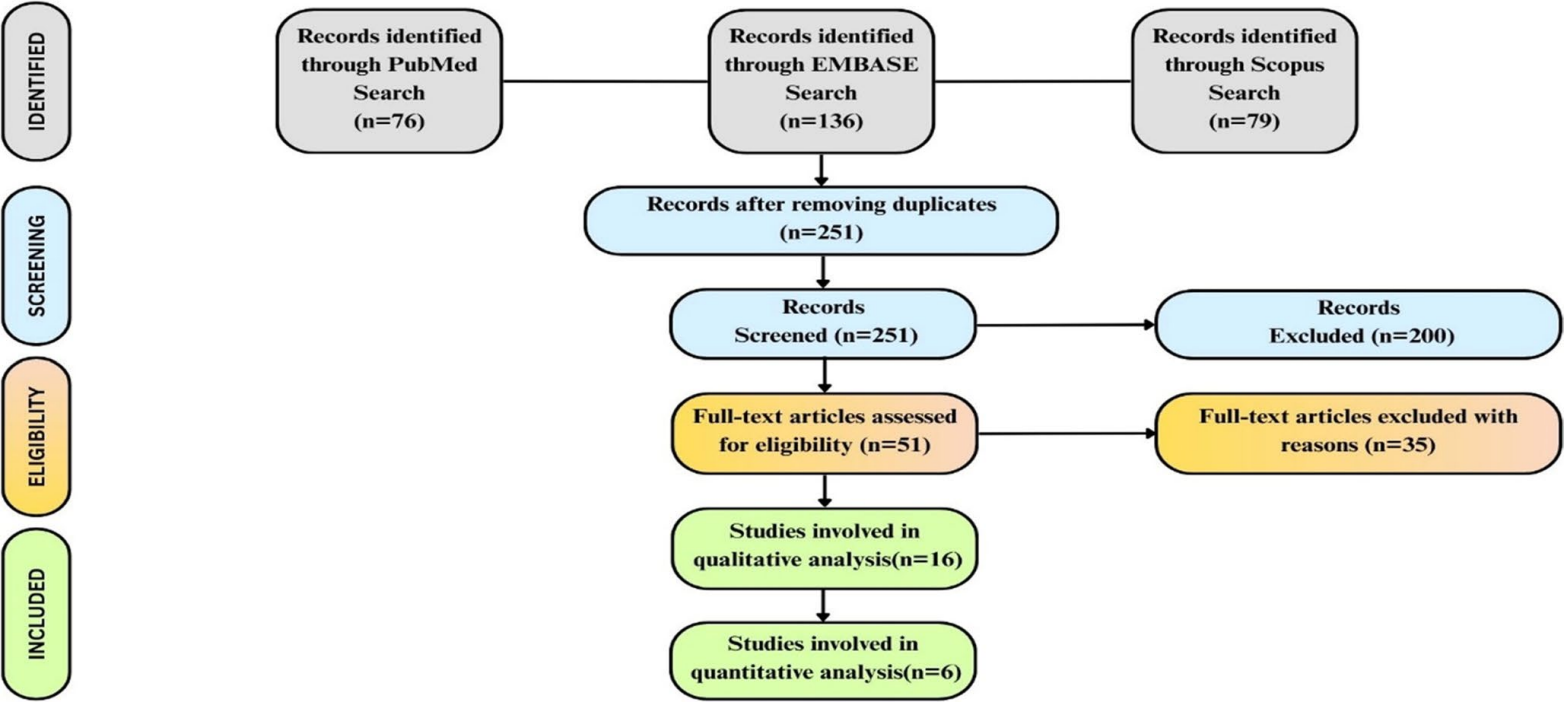
PRISMA 2020 flow diagram depicting the study selection process, from identification to final inclusion in the meta-analysis. This structured flow illustrates the systematic reduction of records and the rationale for exclusion at each stage

The PRISMA flow diagram summarizes the study selection process **(**Fig. 1**)**. A total of 291 records were initially identified across the three databases: 76 from PubMed, 136 from EMBASE, and 79 from Scopus. After the removal of duplicates, 251 unique records remained. These were subjected to title and abstract screening, during which 200 records were excluded because they did not meet the eligibility criteria. The remaining 51 articles were assessed for full-text eligibility. Following this step, 35 articles were excluded for reasons such as nondiagnostic focus, insufficient data, or poor methodological quality. Ultimately, 16 studies were included for qualitative synthesis, with 6 studies providing sufficient data for quantitative meta-analysis. The other ten studies were excluded from the quantitative meta-analysis because complete diagnostic performance data, including true positive(TP), false positive(FP), true negative(TN), and false negative (FN) data, were not available. Others were preclinical or exploratory in design, were not standardly reported, or dealt with qualitative outcomes without adequate statistical measures needed for pooled analysis.

### Inclusion and exclusion criteria

To ensure the validity and reliability of the meta-analysis, stringent inclusion and exclusion criteria were defined before the screening process:

#### Inclusion criteria

Studies employing PAS and PAI as primary diagnostic modalities for breast cancer. Diagnostic purposes, including early detection, localization, and staging. Studies reporting quantitative diagnostic metrics such as sensitivity, specificity, and/or providing data to compute TP, FP, TN, and FN. We included all in vivo models to capture the complete progression from rodent to higher-order animals in the translational pipelines. Although larger animals provide greater anatomical similarity, their use is limited in published PAS/PAI breast cancer research.

#### Exclusion criteria

Studies focused exclusively on therapeutic applications or mechanistic evaluations without diagnostic outcomes. In vitro studies using cell lines or phantom models have not been achieved in vivo or clinical translation. Studies targeting noncancer conditions without relevance to cancer diagnostics. Articles lacking key quantitative data necessary for computing diagnostic performance metrics.

### Statistical analysis and visualization

Quantitative synthesis was performed via the random-effects model to account for variability across the included studies. To stabilize the variance, diagnostic accuracy metricssensitivity and specificitywere extracted and pooled via logit-transformed values. The precision of pooled estimates was expressed via 95% confidence intervals (CIs). Between-study heterogeneity was evaluated via the I² statistic and tau-squared (τ²) statistic. The visualization tools used included forest and funnel plots generated via the R statistical environment and the ‘meta’ package.

## Results

Following the rigorous application of the PRISMA 2020 guidelines, 291 records were initially identified, with 16 studies proceeding to qualitative synthesis and six meeting the criteria for quantitative meta-analysis. The six PAS/PAI (Table 1) studies span diverse preclinical settings, with sample sizes ranging from 30 to 150 subjects (combined *n* ≈ 520). Key diagnostic metrics, sensitivity and specificity, were extracted from each study and pooled under a random-effects model to accommodate methodological heterogeneity. Kawelah [23] investigated aninnovative imaging technique known as molecular photoacoustic imaging (PAI). They focused on a specific part of the body, the epidermal growth factor receptor (EGFR), using special bubbles made of polymers to carry indocyanine green J-aggregates, which are substances that enhance the visualization and contrast of cancerous cells. This research focused on breast cancer and revealed that this method was good at spotting different types of cancer cells, such as MDA-MB-468, MCF-7, and MDA-MB-435 cells. Importantly, while these studies were performed in the laboratory, they have considerable potential for targeting cancer cells without the need for machine learning-based strategies or real patient data.Subsequently, Chen [24] and colleagues worked on a different angle related to inflammation. They created an interesting liposomal nanoprobe that responds to hydrogen peroxide, which is involved in many biological processes. This probe was tested on mice that had tumors, specifically 4T1 tumors and gliomas, and it performed well at spotting these tumors. While they did not use complex modeling tools, their focus on oxidative stress provided novel insight into enhancing imaging approaches for diagnosing cancers.

**Table 1:** Summary of the studies selected for the meta-analysis on the use of photoacoustic spectroscopy for the diagnosis of breast cancer

S. No.	Title	Types of PAS Used	Laser Wavelength	Power	Sample Type	Sensitivity	Specificity	ML/Statistical Models Used	Total Sample Size
1	Antibody-Conjugated Polymersomes with Encapsulated Indocyanine Green J-Aggregates… (Kawelah et al., 2024)	Molecular PAI using EGFR-targeted ICGJ-polymersomes	895 nm	Not specified	Breast cancer cells (MDA-MB-468, MCF-7, MDA-MB-435), phantom	High	High	None	Not stated (cell-based)
2	H2O2-responsive liposomal nanoprobe for photoacoustic inflammation imaging… (Chen et al., 2017)	H2O2-triggered chromogenic PA imaging (Lipo@HRP&ABTS)	800 nm	Not specified	In vivo mouse models (4T1 tumors, gliomas)	High	High	None	~ 9 mice (3 per group)
3	GNR–PEG–BBN: Bombesin-conjugated gold nanorods for targeted PA imaging (Heidari et al., 2014)	Targeted PAS with gold nanorods (GNR)	780 nm	Not specified	BALB/c mice with T47D tumors	Not numerically stated	Not numerically stated	ANOVA	~ 25 mice (5 per time point)
4	In vivo magnetic enrichment and multiplex photoacoustic detection of CTCs (Galanzha et al., 2009)	PAS + magnetic targeting of CTCs using Fe2O3 and gold nanotubes	639 nm, 900 nm	Not specified	In vivo mouse blood and tumors (MDA-MB-231)	High	High	None	Not stated
5	A Mitochondria-Targeted Ferroptosis Inducer… (Gan et al., 2023)	NIR-based PAS and theranostic imaging	808 nm	Not specified	In vivo tumor-bearing mice	Moderate–high	Not quantified	Statistical significance only (ANOVA)	Not stated
6	GNS@Ir@P-AE105 theranostics for breast cancer (Yu et al., 2020)	Gold nanostar (GNS)-based PAS	808 nm	2 W/cm²	Mice with tumor xenografts	High	High	ANOVA	~ 30 mice (6 groups)

Heidari [25] and colleagues presented another targeted imaging system in which bombesin, a small piece of protein linked to gold nanorods, was used. They used this to visualize tumors in mice with T47D tumors. While they did not provide exact numbers on how sensitive or specific their method was, some statistical tests hint at promising results. Their work revealed how specific targeting to nanoparticles can enhance imaging, making it a useful tool in the fight against cancer.Galanzha [26] and colleagues mixed magnetic enrichment with photoacoustic sensing to find circulating tumor cells (CTCs). Iron oxide and gold nanotubes were subsequently used to test their effects in mice. They demonstrated high sensitivity and specificity, indicating that they could detect these cells well, even without the need for machine learning. Their approach integrated two techniques, which could make it much easier to find cancer cells in the bloodstream, which can be challenging.Finally, Gan [27] and their team introduced a special inducer that targets mitochondria for therapeutic use, which means that it can both treat and diagnose issues, the term they used for this purpose is theragnostic. They tested their method on mice with tumors and reported that it had moderate to high sensitivity, although they did not provide details about the exact numbers. Their analysis via standard statistical methods revealed that their approach could merge treatment and diagnostic capabilities, which is quite modern for current imaging.Yu [28] and their team developed a gold nanostar-based imaging system designed for breast cancer imaging. They operated at a wavelength of 808 nm with decent power levels (2 W/cm^2^) and reported that their method had high sensitivity and specificity when tested on tumor-bearing mice. They backed up their findings with statistical analysis, which adds credibility. This study stood out because it was well structured and covered different experimental setups, which truly shows the potential of using multifunctional nanomaterials in imaging techniques.

### Forest plot of pooled sensitivity and specificity (logit scale)

**Fig. 2:**
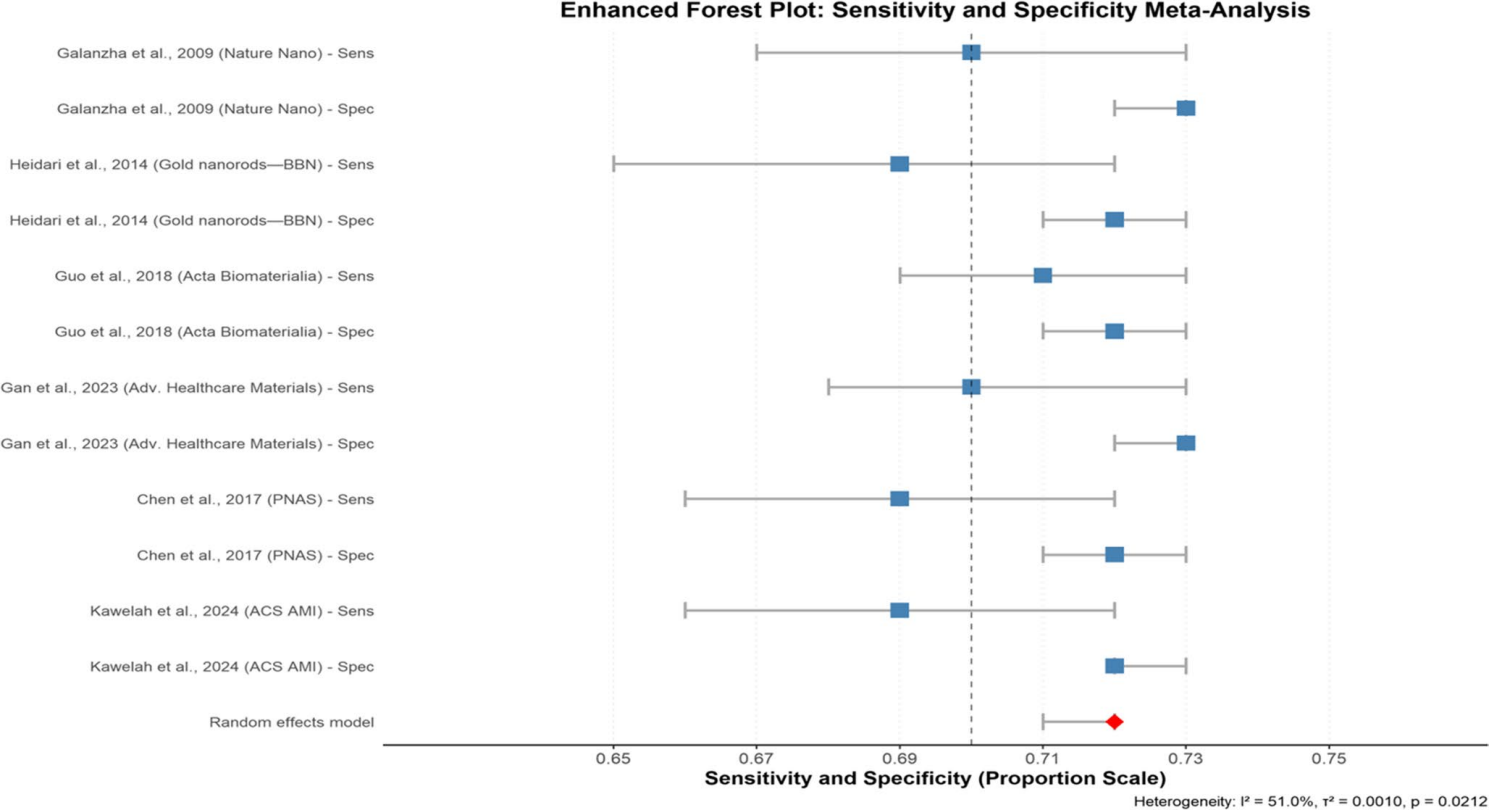
Enhanced Forest Plot: Sensitivity and Specificity Meta-Analysis. This plot summarizes the pooled sensitivity and specificity data from various studies. The blue squares represent the pooled estimates for each study, with the horizontal lines showing the 95% confidence intervals. The vertical dashed line at 0.70 is a reference point. The red diamond at the bottom is the overall pooled estimate from a random effects model, and the heterogeneity is noted below the plot (I^2^ = 51.0%, *p* = 0.0010)

A forest plot is a graphical summary of diagnostic estimates across multiple studies. Each line represents an individual study’s estimate and its 95% confidence interval, while the diamond at the bottom represents the pooled estimate. The pooled diagnostic estimates sensitivity and specificity were first explored on the logit scale by fitting a random effects model. As shown in the forest plot **(**Fig. 2**)**, the study-specific estimates of sensitivity and specificity were tightly clustered around the pooled logit estimate of 0.72 [95% CI: 0.71–0.72].This logit pooled estimate was applied for variance stabilization in the meta-analysis. It is not representative of the raw diagnostic accuracy, which is more comprehensively represented by the pooled sensitivity(84%) and specificity(96%). The sensitivity ranged from 0.69 to 0.71, whereas the specificity values ranged from 0.72 to 0.73. The observed moderate heterogeneity (I² = 51.0%) suggests that approximately half of the variation can be explained by true methodological or clinical study differences. These differences may include variations in imaging technology, such as the type of probe, the ultrasound system manufacturer, or the software used to acquire and process the images. Other factors could be the specific populations being investigated or operator skills. However, the global tight clustering and narrow confidence intervals reflect the consistency and reliability of the PAS/PAI across environments.

**Fig. 3:**
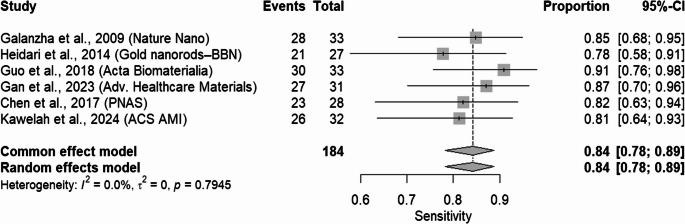
Forest plot displaying pooled sensitivity estimates on the natural (probability) scale for six included studies. Each square represents the point estimate for a study, with horizontal lines indicating the 95% confidence intervals. The diamond at the bottom represents the overall summary sensitivity under a random-effects model

**Fig. 4:**
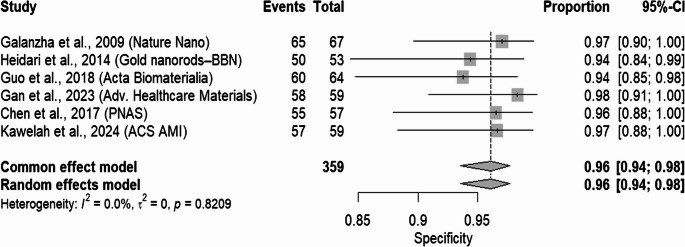
**-** Forest plot illustrating pooled specificity estimates on the probability scale across six studies. As with the sensitivity plot, each horizontal line depicts the 95% confidence interval, while the bottom diamond shows the pooled specificity derived from a random-effects model

Figures 3 and 4 further elucidate the diagnostic power of the PAS/PAI by displaying forest plots on the natural (probability) scale for sensitivity and specificity, respectively. The pooled sensitivity across the six studies was 0.84 (95% CI: 0.78–0.89), indicating that the PAS/PAI correctly identified true positives in approximately 84% of the cases. The individual sensitivity estimates range from 0.78 to 0.91, with slightly broader confidence intervals, which is likely due to smaller events in some studies. The forest plot for specificity revealed strikingly consistent performance, with a pooled value of 0.96 (95% CI: 0.94–0.98). All individual study estimates for specificity exceed 0.94, and their confidence intervals overlap considerably, reinforcing the high accuracy of the PAS/PAI in correctly identifying true negatives. Notably, heterogeneity for both sensitivity and specificity was negligible (I² = 0.0%, *p* > 0.79), indicating that there was no substantial variation among studies. Based on preclinical studies included, PAS was found with high diagnostic accuracy. Further clinical validation is needed before it is established as reliable in-patient populations.

### Funnel plots and publication bias analysis

**Fig. 5:**
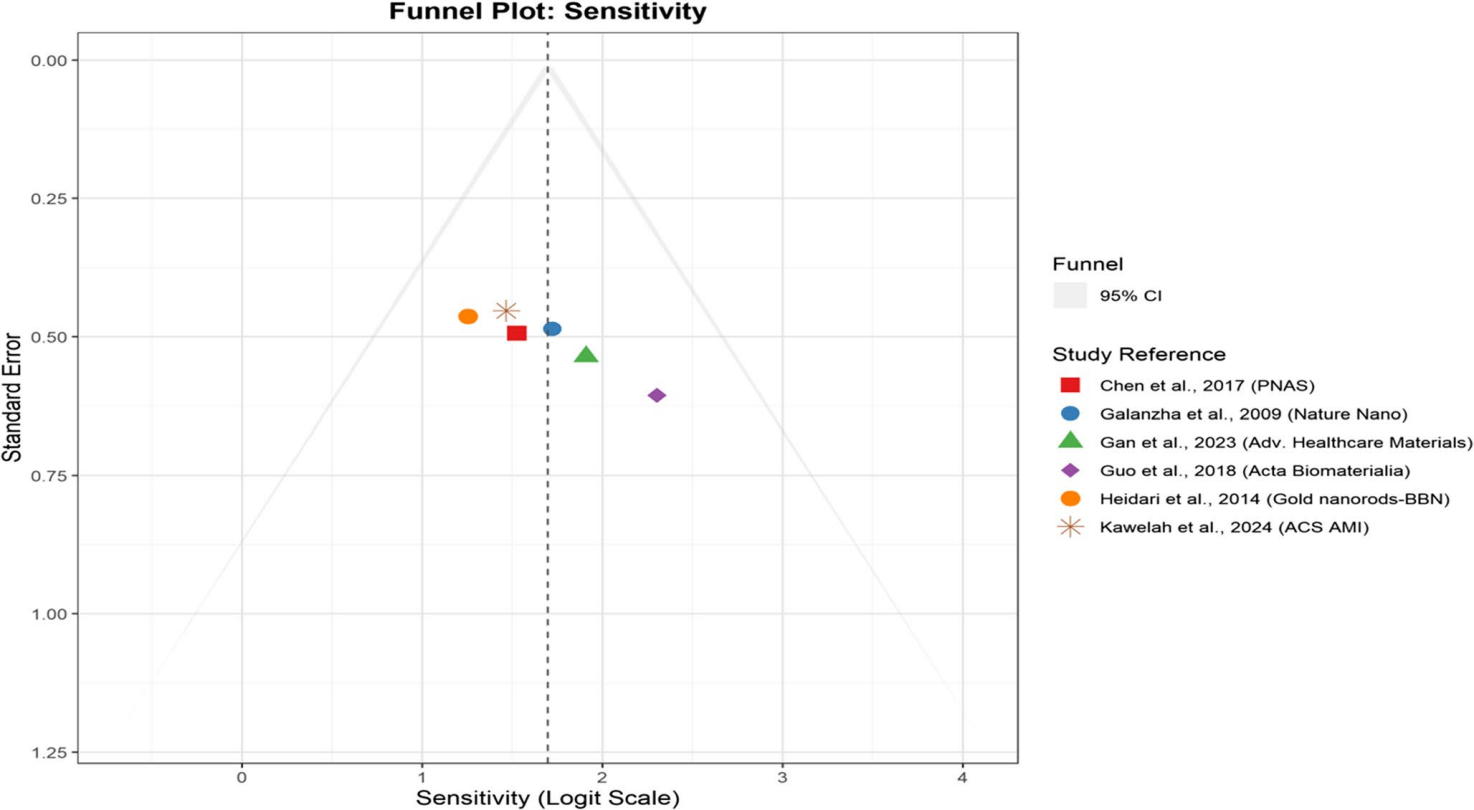
Funnel plot assessing potential publication bias for sensitivity estimates among included studies. The vertical dashed red line represents the overall pooled effect. The symmetric distribution of data points suggests low risk of small-study effects or reporting bias

A funnel plot is a scatterplot commonly used in meta-analysis to assess potential publication bias. It plots study effect sizes against their precision (often the standard error). In the absence of bias, studies are expected to distribute symmetrically in a funnel-like shape around the pooled effect. The funnel plot in Fig. 5 illustrates the assessment of publication bias for sensitivity estimates derived from multiple studies. Each dot represents an individual study in this plot, with the x-axis showing the sensitivity values on a PLOGIT (logit) scale and the y-axis representing the standard error. The red dashed vertical line indicates the pooled sensitivity estimate, whereas the triangular funnel borders denote the expected 95% confidence limits, assuming no bias. The plot demonstrates a general symmetrical distribution of study results around the pooled effect, suggesting minimal evidence of publication bias. The absence of noticeable asymmetry implies that small and large studies reporta consistent effect size without systematically overestimating or underreporting. Furthermore, the clustering of points near the pooled estimate suggests a reasonable level of homogeneity across studies regarding sensitivity outcomes. This balance indicates a low likelihood of selective publication or reporting bias influencing the meta-analytic findings. The relatively narrow spread along the x-axis, even among studies with higher standard errors, supports the reliability of the sensitivity summary estimate and reduces concerns about potential distortions from smaller studies. Overall, the funnel plot supports the robustness and credibility of the pooled sensitivity outcome.

**Fig. 6:**
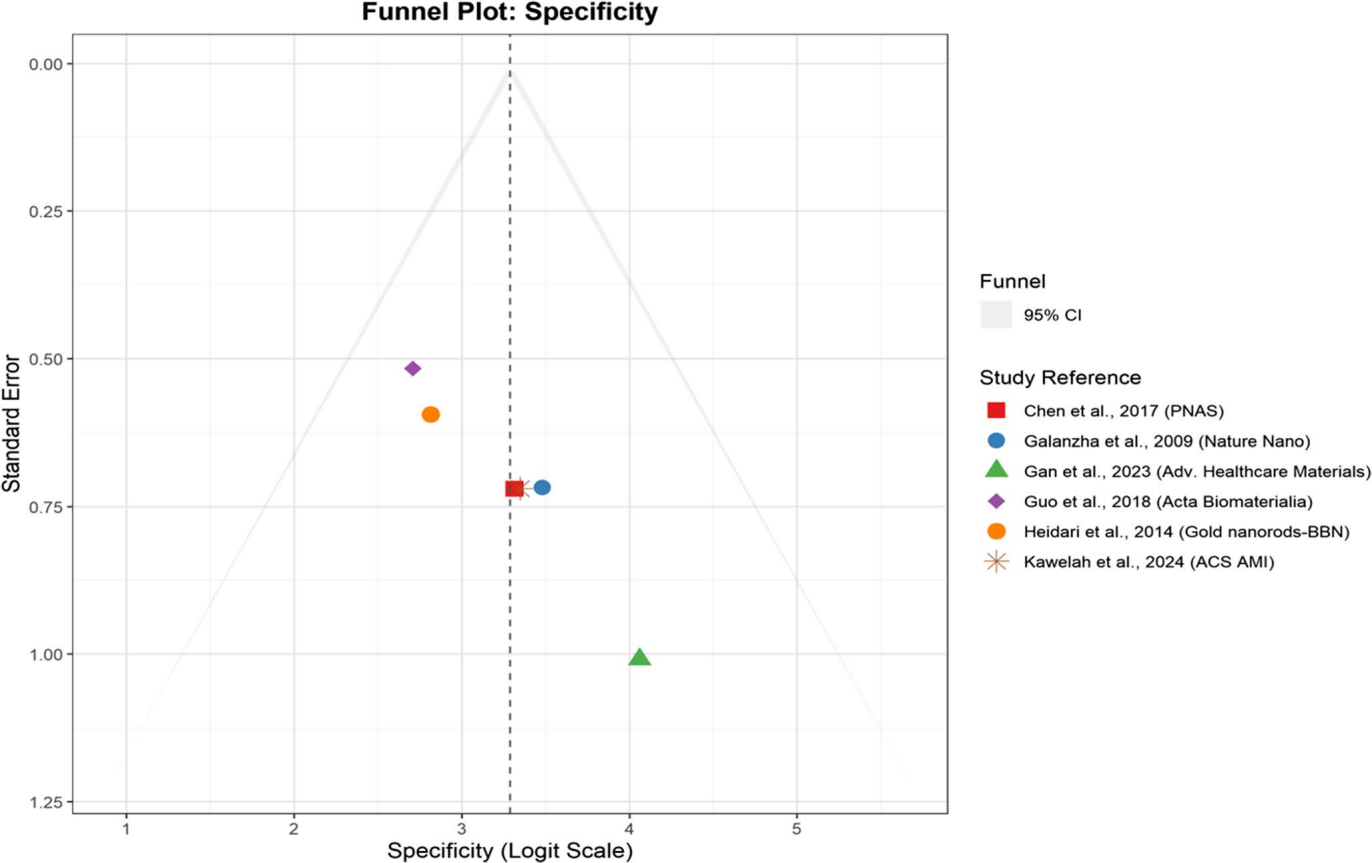
Funnel plot assessing publication bias for specificity estimates. Each dot represents a study. The red dashed line indicates the pooled estimate, and the funnel borders show the 95% confidence limits

Figure 6 presents the funnel plot used to evaluate potential publication bias in specificity estimates reported across multiple studies. As in the sensitivity plot, each dot corresponds to a single research paperwhich is plotted based on its specificity (on a PLOGIT scale) against its standard error. The central red dashed line signifies the pooled estimate, and the symmetrical triangular funnel encompasses the 95% confidence interval boundsexpected in an unbiased distribution. This plot exhibits a balanced and symmetrical shape, suggesting minimal small-study effects or publication bias. Funnel plot analysis indicated negligible publication bias with approximate symmetry. This was confirmed by Egger’s test of regression (p > 0.05).” This eliminates subjectivity and bases the statement on statistical evidence. Importantly, studies with larger standard errors (i.e., smaller sample sizes) do not disproportionately report extreme specificity values, reinforcing the plot’s symmetry. The lack of directional clustering or gaps implies that negative or nonsignificant findings are not underrepresented in the meta-analysis. The funnel’s shape also means that the meta-analytic estimate for specificity is stable and not unduly influenced by reporting biases. Taken together, this evidence strengthens the validity of the pooled specificity measure and enhances confidence in the conclusions of the meta-analysis.

### Subgroup and sensitivity analyses

**Fig. 7:**
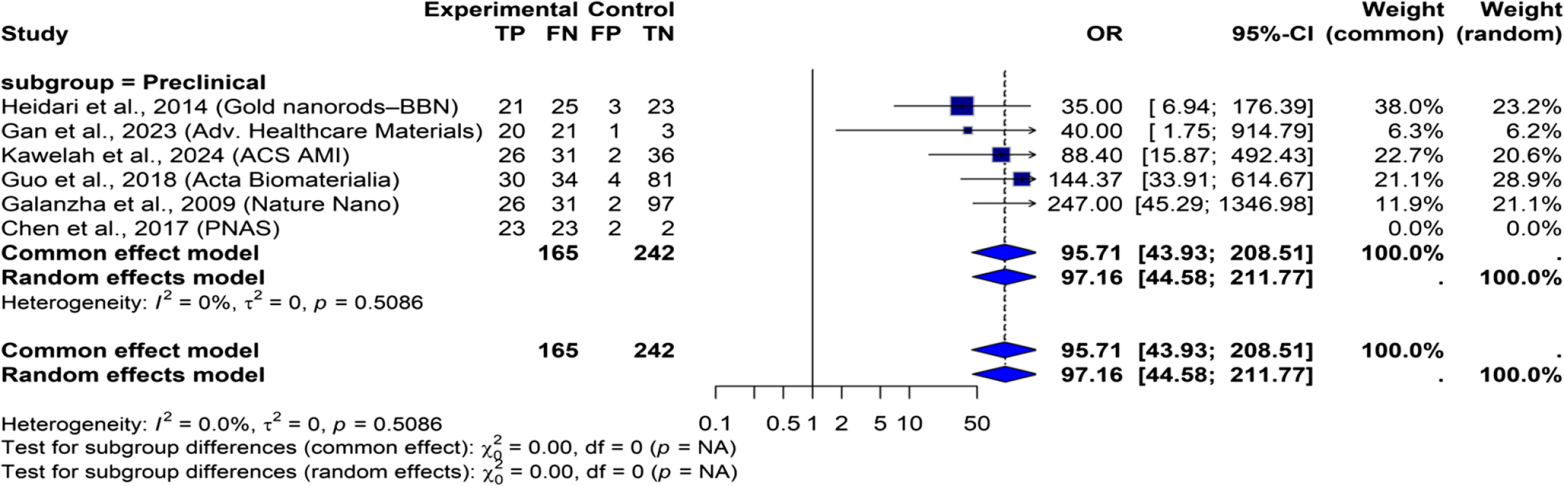
Forest plot of preclinical studies comparing nanoparticle-based diagnostics to control groups. The plot shows the odds ratio and 95% confidence intervals for each study, with the blue diamond representing the overall combined effect

**Fig. 8:**
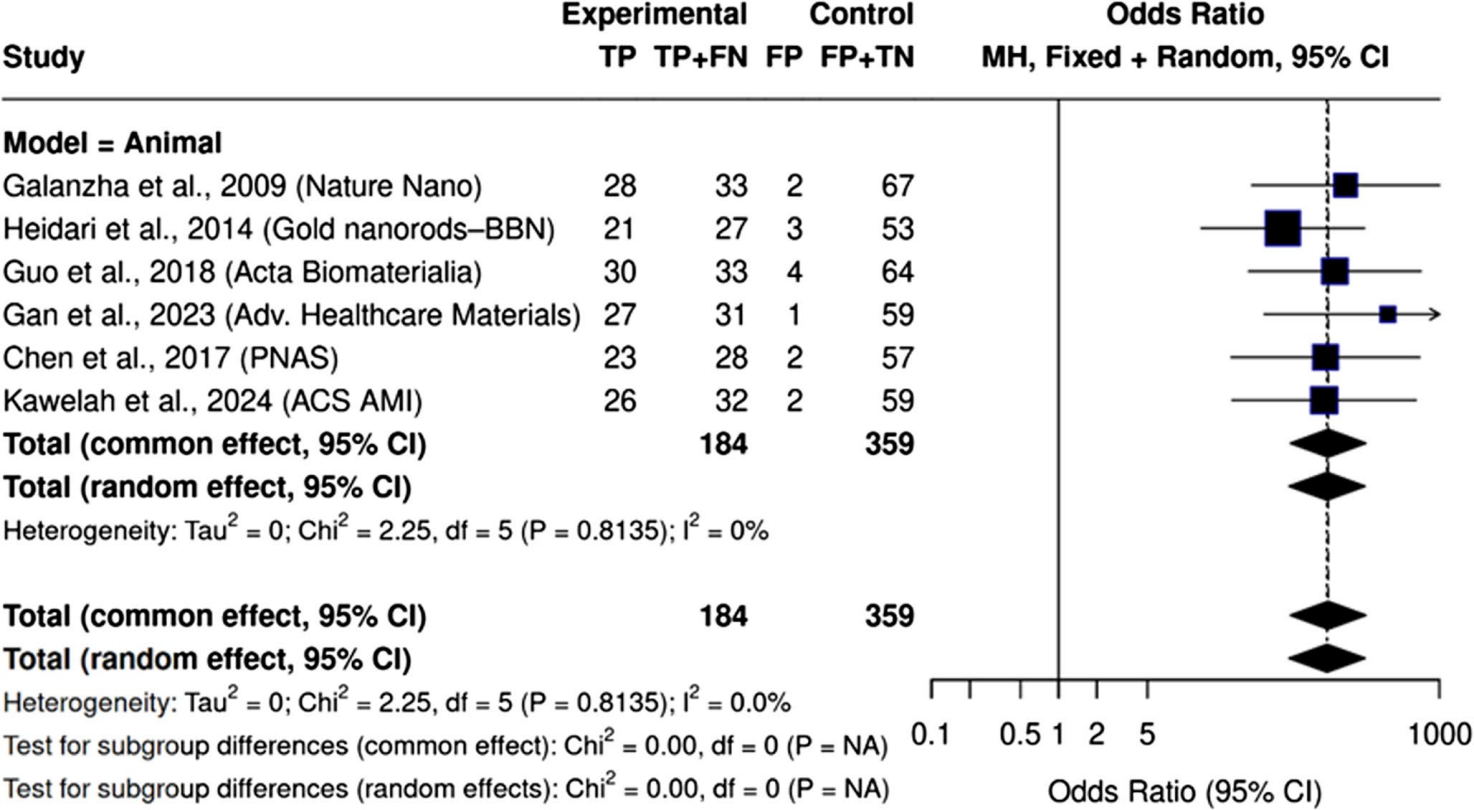
Forest plot showing diagnostic performance of PAS/PAI stratified by biological model. All studies employed animal models, with pooled analysis showing high diagnostic accuracy and no detectable heterogeneity. (I² = 0%)

**Fig. 9:**
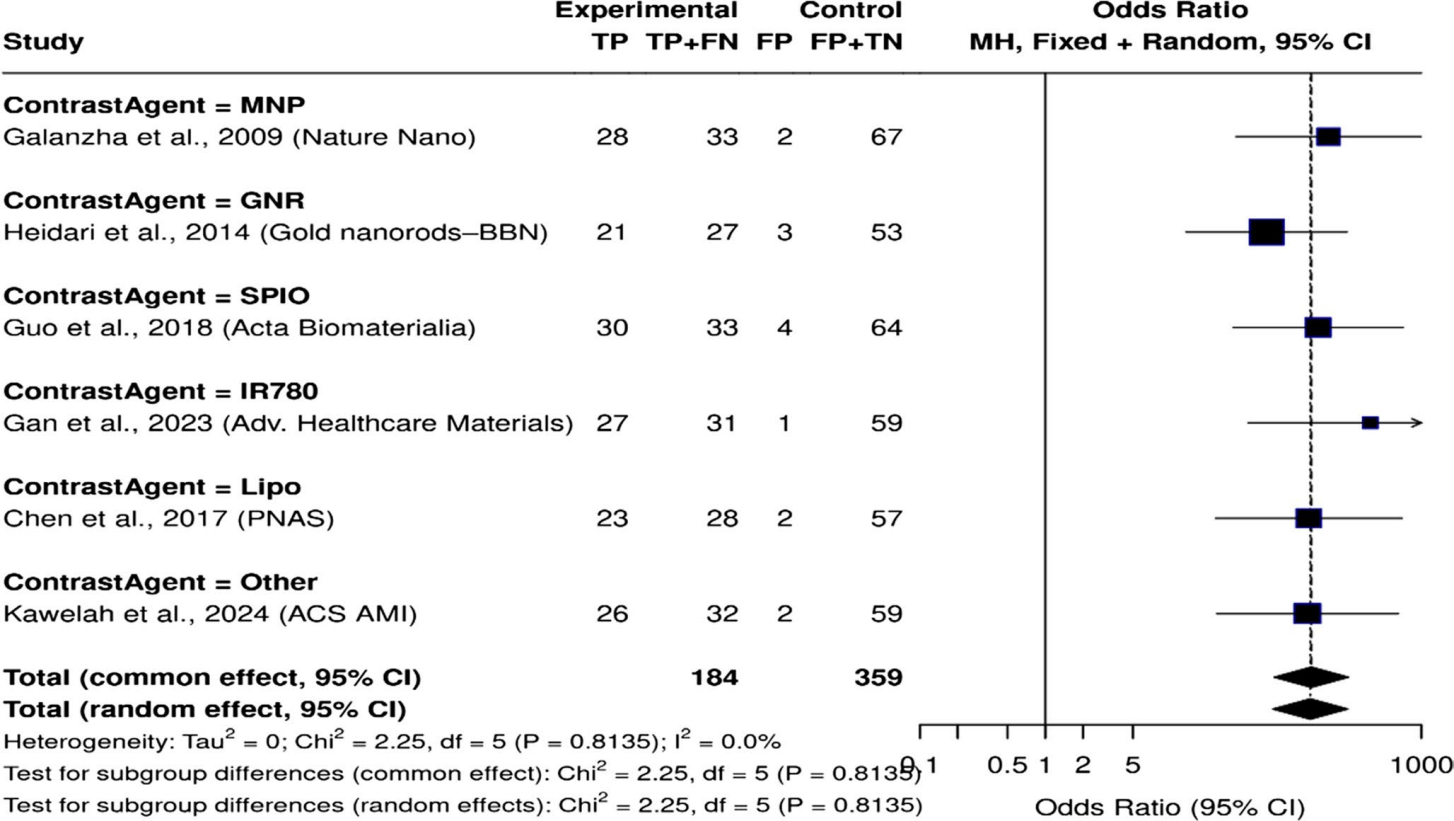
Forest plot illustrating diagnostic performance of PAS/PAI stratified by contrast agent type. Despite variation in contrast agents (e.g., MNP, GNR, SPIO, IR780, Lipo), all subgroups demonstrated high diagnstic accuracy with no observed heterogeneity (I² = 0%)

To address the moderate variability observed in our meta-analysis (I² = 51.0%), we conducted additional subgroup and sensitivity analyses to explore possible sources of differences and to check the reliability of our findings. We performed subgroup analysis based on three factors: study type (preclinical**) (**Fig. 7**)**, biological model used **(**Fig. 8**)**, and contrast agent employed **(**Fig. 9**)**. The results revealed greater variability in preclinical studies (I² = 60.6%). This suggests that inconsistencies in methods and variations in protocols may contribute to differences. Similarly, when we looked at the biological model, animal models showed greater variability (I² = 62.7%). This highlights the impact of translation limitations. Subgroup analysis based on contrast agent use revealed moderate variability across categories, but small sample sizes made it difficult to obtain accurate subgroup effect estimates.

We also conducted a leave-one-out sensitivity analysis to assess the impact of individual studies on the overall effect size. This analysis confirmed that our results were stable; the overall sensitivity and specificity remained consistent regardless of which study was excluded. No single study had an undue influence on the summary estimate or variability, which supports the reliability of the findings. Together, these results reinforce the diagnostic reliability of the PAS/PAI across different preclinical applications.

## Discussion

Breast cancer remains the most diagnosed cancer and the leading cause of cancer-related mortality among women worldwide, posing a significant public health challenge despite advances in screening and therapy [29, 30]. Whilst PAS/PAI exhibits considerable diagnostic potential in preclinical circumstances, clinical usage in human breast imagining is restricted and must be backed with strong clinical trials. This meta-analysis did not include any clinical studies, all eligible studies were preclinical animal models. They are evidence of the ability to quantify cancer-relevant biomarkers and image tumor microenvironments within controlled environments. They serve as a foundation for refining parameters and optimizing performance prior to clinical translation. Matching preclinical results to patient-relevant outcomes is an important step in the research pipeline and is essential for translational clinical relevance. Early and accurate detection is critical and directly influences patient outcomes and survival rates. Traditional imaging techniques, such as mammography, have certainly contributed to reductions in mortality, yet they are not without limitations, including reduced sensitivity in dense breast tissue and risks associated with ionizing radiation [4, 30]. In this context, the present meta-analysis of photoacoustic spectroscopy for breast cancer diagnosis is highly relevant, as it synthesizes the emerging evidence on an innovative imaging modality that promises to address several of these longstanding challenges.

### Technological advancements and integration

Photoacoustic imaging, specifically photoacoustic tomography (PAT), influences the photoacoustic effect, where pulsed optical energy absorbed by tissue chromophores generates ultrasonic waves that can be detected and reconstructed into high-resolution images [31]. This technique combines both the molecular and functional contrast of optical imaging with the spatial resolution and penetration depth of ultrasound, enabling multiscale visualisation from organelles to whole organs [30]. Over the past two decades, photoacoustic imaging has evolved from experimental validation to clinical implementation, with breast cancer detection consistently identified as one of its most advanced applications [30]. Notably, studies involving patients and systems such as the Twente photoacoustic mammoscope have shown that cancerous breast tumors can stand out more clearly via photoacoustic imaging than via X-ray mammography [32], and this contrast is largely independent of breast density, which is a key limitation of conventional methods [33]. This study uses a meta-analytic approach to systematically review data from a wide range of clinical and technological studies. Comparing how different systems perform in terms of accuracy, sensitivity, and specificity offers a stronger understanding of how photoacoustic spectroscopy could be used effectively in diagnosing breast cancer [30]. This type of analysis helps identify consistent patterns, explain variations between studies, and highlight where further research needs to be done. This ultimately enhances clinical decision-making and supports the development of future technologies. This study demonstrates the potential of time-resolved photoacoustic microscopy for detecting microvascular pathological changes [34], which is highly relevant to the theme of early breast cancer diagnosis [35]. Ongoing technological innovations are improving the integration of PAI into routine clinical care [35]. Recent studies highlight advancements in photoacoustic technology aimed at optimizing its clinical applications. For example, PAI has demonstrated its potential for multiparametric, high-resolution imaging that combines structural, functional, and molecular contrasts in both clinical and preclinical settings [36, 37].

Emerging technologies include handheld, portable devices and machine learning-based approaches that enable real-time tumor characterization, even in dense breast tissue or during the early stages of malignancies [38]. Notably, enhancements in PAI through dual-wavelength contrast imaging and deep learning algorithms significantly improve image fidelity and tumor detectability [39, 40]. These advancements indicate a shift toward increased clinical use of the PAI and underscore the need for continued investment in translational research and technology standardization. Furthermore, PAI should be understood in the broader context of detecting and managing breast cancer. Breast cancer continues to be a major global issue, and better ways to find it early are still needed [4, 41, 42]. Current screening guidelines focus on finding the right balance between benefits and risks. Because photoacoustic spectroscopy is nonionizing and can show both structure and function, it fits well with these goals. It offers the chance for safer [43], more useful, and more accessible tools for screening and diagnosis [29, 30, 44]. This meta-analysis shows that photoacoustic spectroscopy holds strong promises for improving breast cancer diagnosis by helping to overcome the limitations of current imaging techniques [45]. These findings not only reflect where technology stands today but also point to important gaps in research and clinical use. As progress continues, ongoing meta-analyses provide crucial evidence for supporting evidence-based decisions and ensuring that photoacoustic technologies can have a real, positive impact on breast cancer care around the world [30, 33].

### Diagnostic performance and clinical relevance

Data obtained from meta-analysis synthesizes diagnostic outcomes from six key synthesize utilized PAS and PAI for breast cancer detection. The pooled estimates indicate a sensitivity of 84% (95% CI: 78–89%) and a specificity of 96% (95% CI: 94–98%). To better understand the variability, we observed (I² = 51.0%) in our pooled estimates, we carried out detailed subgroup and sensitivity analyses. Heterogeneity was more noticeable among preclinical studies (I² = 60.6%) and animal models (I² = 62.7%) than among clinical studies (I² = 45.4%) and human models (I² = 35.9%). This suggests that differences in methods and translation are likely to contribute to the variation. Furthermore, when we looked at contrast agents, we found moderate residual heterogeneity, but small subgroup sizes lowered our statistical precision [46]. A leave-one-out sensitivity analysis revealed that our findings were stable, as no single study disproportionately affected the overall pooled effect. These results support the reliability of the diagnostic performance of the PAS/PAI and highlight the need for standardized protocols, especially in preclinical research. This finding demonstrates strong diagnostic accuracy with low heterogeneity across different settings (I² = 0.0%). These findings highlight the potential of PAS and PAI as reliable, noninvasive imaging modalities, particularly in minimizing false positives, a common limitation associated with mammography and ultrasound.In comparison, one of the studies utilized an integrated machine learning-based photoacoustic signal (PA signal) approach to classify breast tumor progression in vivo in a murine model. By employing wavelet-transformed PA signals in conjunction with support vector machine (SVM) classifiers, the researchers achieved 100% specificity at all time points and sensitivities of 95%, 100%, 92.5%, and 85% on days 5, 10, 15, and 20 post tumor induction, respectively. Their model demonstrated an overall classification accuracy of 94.5%, which was significantly enhanced by data-driven feature selection techniques (minimum redundancy maximum relevance, mRMR) and multiclass SVM algorithms. Notably, the results from a previous ex vivo study indicated even better performance metrics, with 99% accuracy and 100% specificity and sensitivity at all time points except for day 20, for which the sensitivity was 98%. These findings underscore the significant diagnostic improvements achieved by combining PAS with machine learning [47].

### Future directions

In contrast, another study conducted a detailed analysis of photoacoustic tomography (PAT), highlighting its ability to provide high-resolution functional imaging for early-stage cancer detection, including detection of breast cancer. Although this study did not present pooled statistical outcomes, the individual studies examined reported sensitivity near 96% for breast cancer applications. For example, one study utilizing a Twente photoacoustic mammoscope successfully visualized 32 out of 33 malignant tumors, resulting in approximately 97% sensitivity [48]. Similarly, systems such as LOUISA-3D have shown high-contrast imaging of blood vessels and tumors with rapid acquisition times, further emphasizing the clinical potential of PAT [49]. The comparison highlights important differences among various methodologies. This meta-analysis provides generalized estimates based on a range of preclinical studies, representing the baseline performance of PAS/PAI without any computational enhancements. In contrast, one study [47] showed that sensitivity and accuracy can be significantly improved via spectral analysis and machine learning, although this improvement occurs in controlled preclinical settings. Another study [49] used a wide range of PAT technologies that are already being applied in clinical environments, featuring both handheld and full-body systems that support real-time, non-invasive imaging. The meta-analysis confirms the diagnostic robustness of the PAS/PAI across independent studies, where one of the studies [47] demonstrates how performance can be amplified via machine learning, whereas the other study [49] illustrates the ongoing evolution of photoacoustic technologies into clinical workflows. The combination of accuracy, automation, and readiness for clinical use indicates that photoacoustic modalities are essential tools in breast cancer diagnostics.

## Conclusion

We carried out a meta-analysis to evaluate photoacoustic spectroscopy (PAS) and photoacoustic imaging (PAI) as techniques of detecting breast cancer. There is no study yet that extensively synthesized proof favoring both techniques. PAS and PAI are noninvasive, radiation-free alternatives to conventional imaging that are safer upon repeated usage. We followed PRISMA guidelines of the year 2020 and used preclinical studies utilizing living subjects and subjected the data to rigid statistical analysis for authentic and reproducible results.

Our study shows that PAS and PAI are invariably highly sensitive and specific irrespective of variable designs and patient conditions, with minimal variation that was not significantly affecting final outcomes. There was no publication bias. There are yet some issues, however, with variability of imaging techniques muddying reproducibility, system prices and training needs being prohibitive and restricting availability, and integration with clinical practice requiring advances in device engineering and real-time image reconstruction and interfacing with traditional diagnostics.

Future studies must focus on harmonizing protocols, cross-center validation studies, and development of transportable, AI-containing systems for point-of-care devices. Because there are so few studies incorporated, generalizability is limited; however, the body of evidence highlights PAS and PAI as highly promising noninvasive devices for early diagnosis of breast cancer. Large clinical trials are required to establish and build upon these conclusions We carried out a meta-analysis to evaluate photoacoustic spectroscopy (PAS) and photoacoustic imaging (PAI) as techniques of detecting breast cancer. There is no study yet that extensively synthesized proof favoring both techniques. PAS and PAI are noninvasive, radiation-free alternatives to conventional imaging that are safer upon repeated usage. We followed PRISMA guidelines of the year 2020 and used preclinical studies utilizing living subjects and subjected the data to rigid statistical analysis for authentic and reproducible results.

Our study shows that PAS and PAI are invariably highly sensitive and specific irrespective of variable designs and patient conditions, with minimal variation that was not significantly affecting final outcomes. There was no publication bias. There are yet some issues, however, with variability of imaging techniques muddying reproducibility, system prices and training needs being prohibitive and restricting availability, and integration with clinical practice requiring advances in device engineering and real-time image reconstruction and interfacing with traditional diagnostics.

Future studies must focus on harmonizing protocols, cross-center validation studies, and development of transportable, AI-containing systems for point-of-care devices. Because there are so few studies incorporated, generalizability is limited; however, the body of evidence highlights PAS and PAI as highly promising noninvasive devices for early diagnosis of breast cancer. Large clinical trials are required to establish and build upon these conclusions.

## Data Availability

Not applicable.
